# Accuracy and reliability of mandibular digital model superimposition based on the morphological characteristics of vessels in extraction adult patients

**DOI:** 10.1186/s12903-023-03836-9

**Published:** 2024-01-24

**Authors:** Yaozheng Hu, Mengyu Zheng, Jin Chen, Chenlin Guo, Jianming Chen

**Affiliations:** 1 Department of Orthodontics, School and Hospital of Stomatology, Guangdong Engineering Research Center of Oral Restoration and Reconstruction, Guangzhou Medical University, Guangzhou, China; 2 Guangzhou Key Laboratory of Basic and Applied Research of Oral Regenerative Medicine, Guangzhou, China

**Keywords:** Superimposition, Mandibular, Digital models, CT, Vessels

## Abstract

**Background:**

This study aimed to validate the availability of superimposing full-color mandibular digital models (DMs) by the morphological characteristics of vessels in extraction adult patients.

**Methods:**

Twenty-eight adult patients were included, and their DMs were superimposed with pre- and posttreatment cone beam computed tomography (CBCT) and the morphological characteristics of lingual vessels. The measurements of each tooth were compared under the same coordinate system.

**Results:**

The *ICC* results displayed exceptional agreement in intra- and interrater assessments, with scores exceeding 0.891 in the crown for intrarater agreement and scores surpassing 0.888 in the crown for interrater agreement. Furthermore, no statistically significant differences were found in the 2 superimposition methods (*P* > 0.05).

**Conclusion:**

The morphological characteristics of vessels under the mucogingival junction in the lingual side of mandible of are stable enough for the superimposition of mandibular DMs in the adult patients undergo orthodontic treatment with premolars extraction.

## Background

Dento-maxillofacial function, stability, aesthetics and health can be achieved by orthodontic tooth movement. Therefore, orthodontists must assess three-dimensional tooth position accurately and effectively. Several methods have been reported to evaluate tooth movement: plaster model, radiography such as lateral cephalogram and cone beam computed tomography (CBCT). However, plaster model casts have gradually declined because of their breakable characteristic [1, 2]. The lateral cephalograms reveal the 2-D tooth movement and the airway with defects such as magnification and distortion in the image [3, 4]. As for CBCT images, which are now widespread in the clinic for presenting 3-D images of dental-maxillofacial anatomy without image distortion [5–7], it has been reported that CBCT is capable of evaluating tooth movement through voxel-based superimposition [8–11]. However, for orthodontists, it violates the principle of as low as reasonably achievable (ALARA) to take CBCT repeatedly in a short period only to evaluate tooth movement.

3-D intraoral scanning has become widespread for diagnosis and treatment in dental clinics. This technology allows recording teeth and portions of periodontal tissue without exposing the patient to harmful radiation [12, 13]. The intraoral structures are saved as digital data, which are unbreakable and available for 3-D measurement. Some studies have reported that the palatal rugae and palatal vault could remain stable during orthodontic treatment and be the reference region in maxillary DMs superimposition [14–18]. Regarding mandibular DMs, few anatomical structures can be recorded by a scanner. Therefore, some studies have superimposed mandibular DMs indirectly by CBCT registration or occlusion of the maxilla and mandible [19–21]. It is difficult to superimpose the mandibular DMs accurately, even though some researchers considered the mandibular torus could be stable during orthodontic treatment [22]. However, the torus is not prevalent in every patient. Therefore, it is urgent to find a method to superimpose mandibular DMs efficiently and accurately.

Some researchers have confirmed that the branches of vessels in the fundus from different periods are available for image superimposition and further instruct the clinical diagnosis [23, 24]. Thus, we suggested the hypothesis that the morphological characteristics of vessels could also be a potential reference for superimposing mandibular DMs. Our study aims to establish the viability of superimposing full-color mandibular digital models (DMs) by evaluating vessel morphological features in adult patients who have completed orthodontic treatment with premolars extraction. The outcome of this evaluation will be compared to that of CBCT voxel-based DM superimposition, regarded as the benchmark standard.

## Methods

All patients were collected from the Department of Orthodontics with the following inclusion criteria: (1) adult patient; (2) CBCT images and DMs data were preserved perfectly; (3) patient finished the orthodontic treatment with 4 premolars extraction. Moreover, the exclusion criteria were as follows: (1) patients with missing teeth (excluding the third molars); (2) patients who had undergone orthognathic surgery; (3) patients with severe periodontal diseases. Twenty-eight adult patients (2 males, 26 females, 22.54 ± 2.19 years) were included, and their CBCT images and DM data at 2 different time points, pretreatment (T1) and posttreatment (T2), were obtained as well. The mean duration of treatment was 26 months.

The department of the medical image acquired the CBCT images with Carestream Health CS 9300 (Carestream Health Inc., Rochester, NY), and the parameters were as follows: Field of View (FOV) 170 mm* 135 mm; 0.3 mm^3^ pixel size; 90 kVp; 4 mA; and 12–28 seconds of exposure. All patients were required to maintain the intercuspal position with the head fixed.

The department of orthodontics collected the DMs data with TRIOS 3D intraoral scanner (v1.3.4.7, 3-Shape Inc., Copenhagen, Denmark). All scanning operations were conducted under the guidance of the manufacturer. The data we collected were further processed through the procedures shown as follows.

**Fig. 1:**
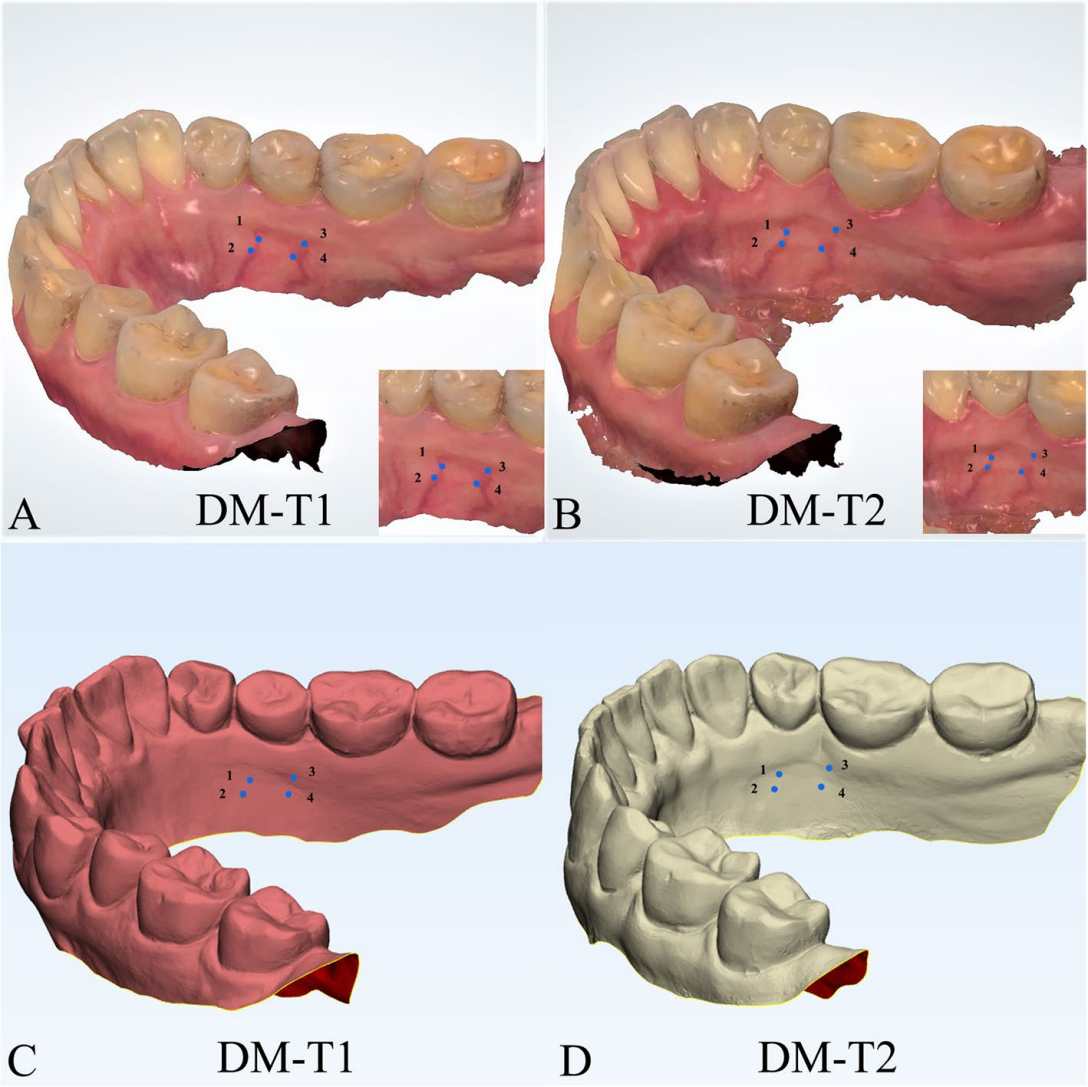
Marker placement. Large boxed areas show high-magnification views. **A** Pretreatment DMs (DM-T1) with markers. **B** Posttreatment DMs (DM-T2) with markers. **C** DM-T1 in standard triangulated language format (Red). **D** DM-T2 in standard triangulated language format (yellow)

**Fig. 2:**
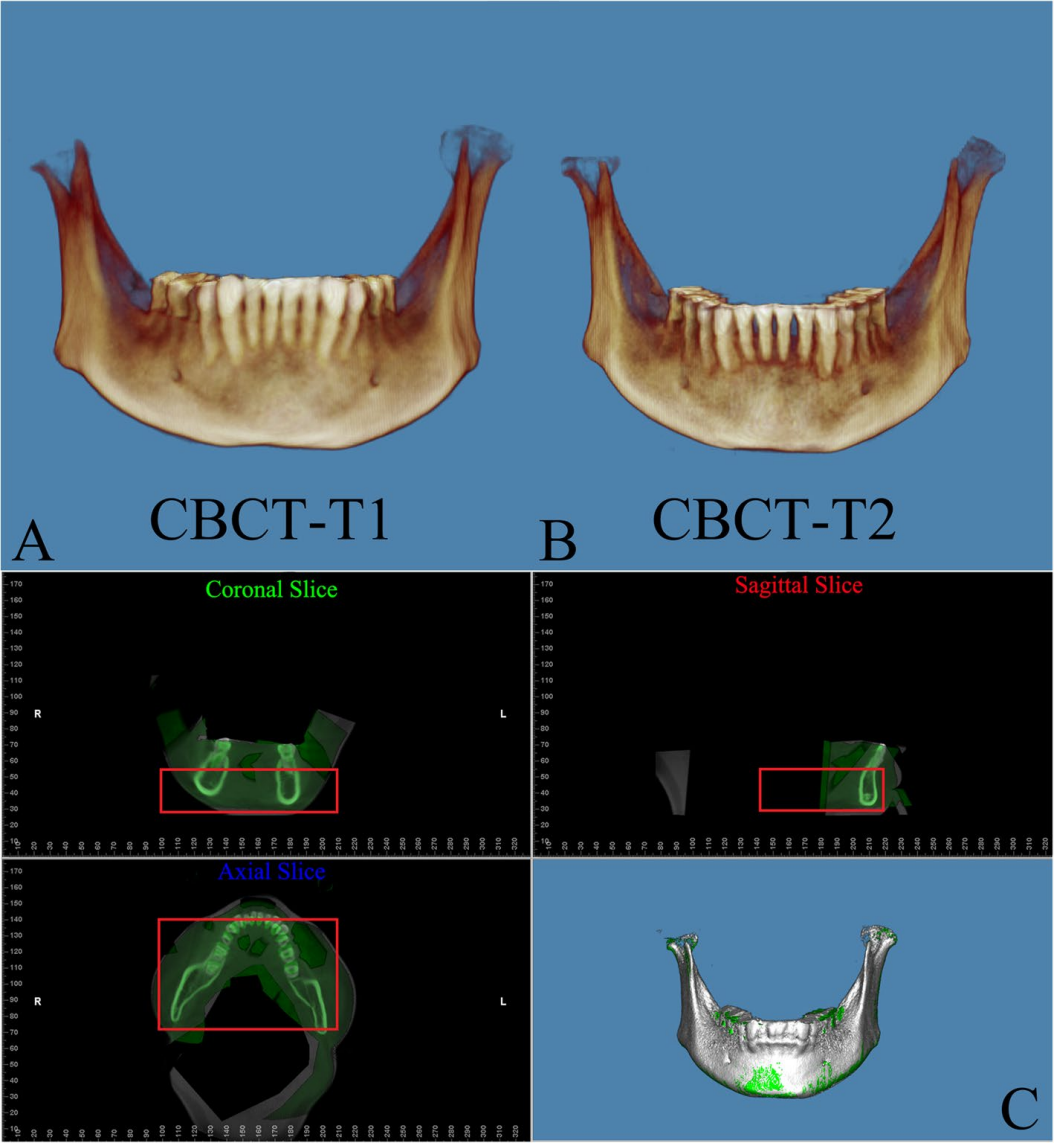
**A** Pretreatment CBCT (CBCT-T1). **B** Posttreatment CBCT (CBCT-T2). **C** Voxel-based CBCT registration (CBCT-T1: white, CBCT-T2: green, the red box displays the chosen reference area)

**Fig. 3:**
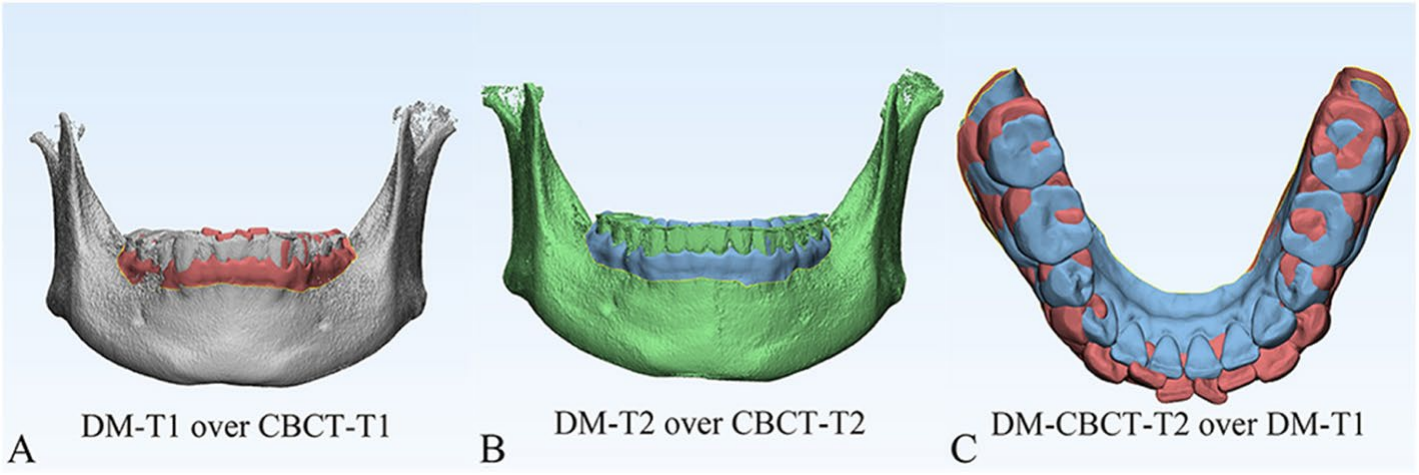
**A** Settle the position of DM-T1. **B** Registered the DM-T2 to CBCT-T2. **C** Acquisition of the CBCT-based DMs superimposition, DM-T1 (red) and DM-CBCT-T2 (blue)

**Fig. 4:**
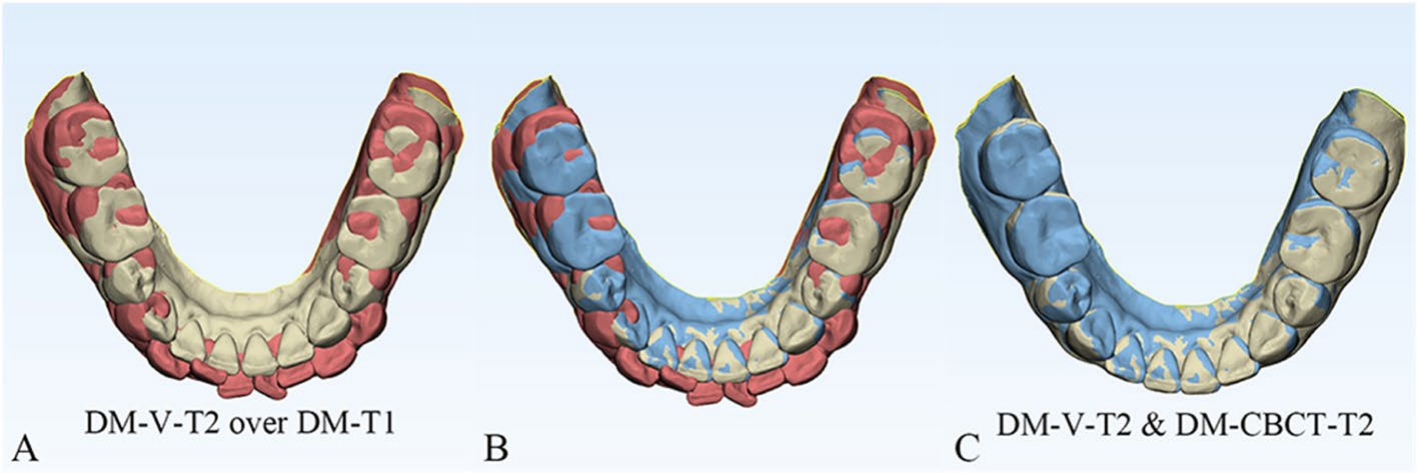
**A** Vessel-based DMs superimposition, registration of DM-V-T2 (yellow) to DM-T1 (red). **B** DM-T1 (red) and DM-CBCT-T2 (blue) and DM-V-T2 (yellow). **C** Top views of 2 methods of superimposition

**Fig. 5:**
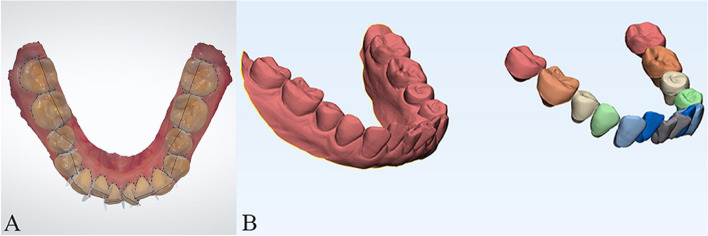
**A** Segmentation. **B** Transferring the scanning models to the dentition model

**Fig. 6:**
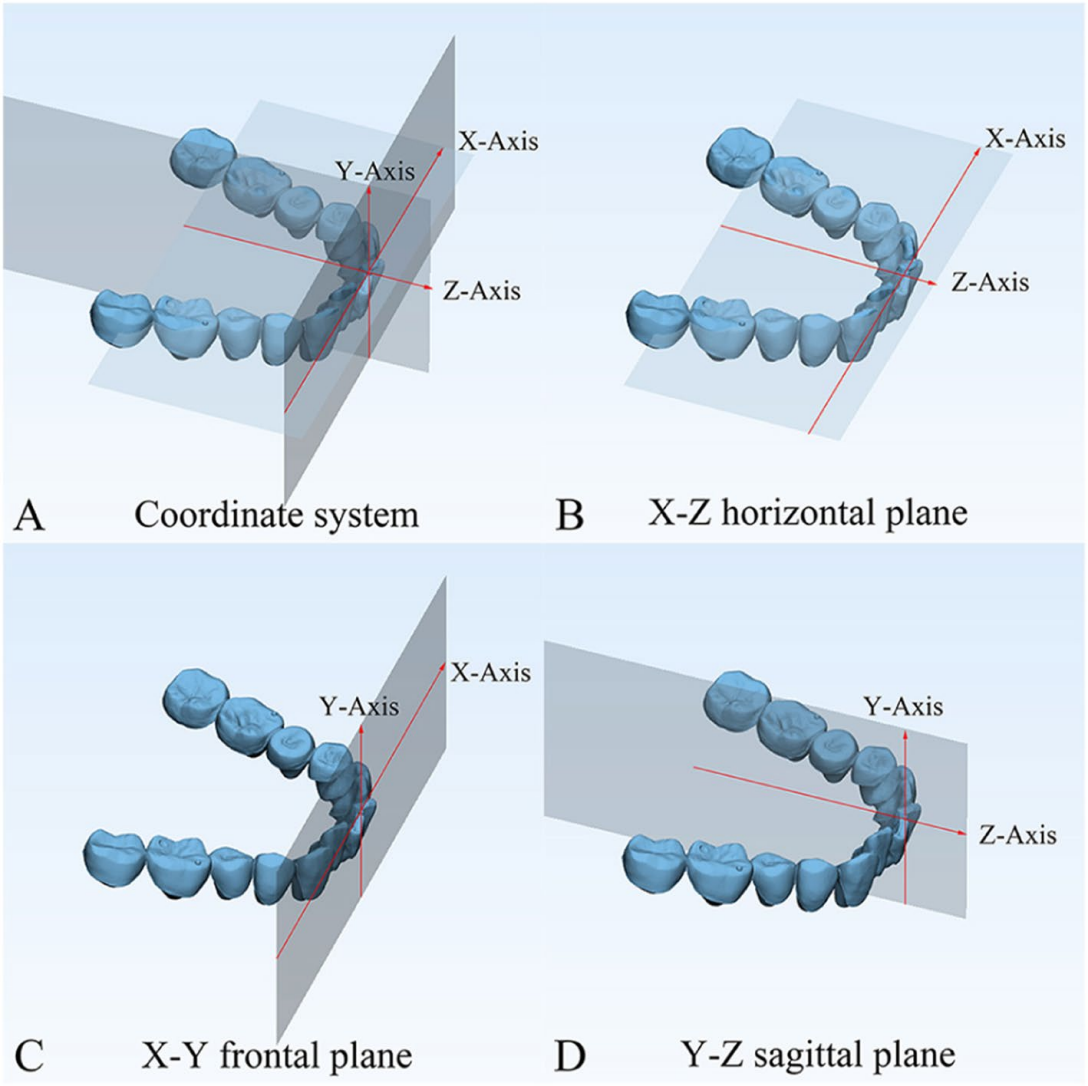
**A** Coordinate system of dentition models. The X-axis, a line through the midpoint of bilateral incisors and parallel to the line connecting bilateral first molar mesial-buccal cusps, stands for the horizontal direction. The Y-axis was perpendicular to the X-axis and through the midpoint of bilateral incisors, which stands for the vertical direction. The Z-axis was perpendicular to the X-axis and Y-axis through the midpoint of the bilateral incisors, which represents the sagittal direction. **B** X-Z horizontal plane, constructed on the mandibular oral plane by 3 spheres. **C** The X-Y frontal plane was perpendicular to the horizontal plane. **D** The Y-Z sagittal plane was perpendicular to the horizontal plane and frontal plane

**Fig. 7:**
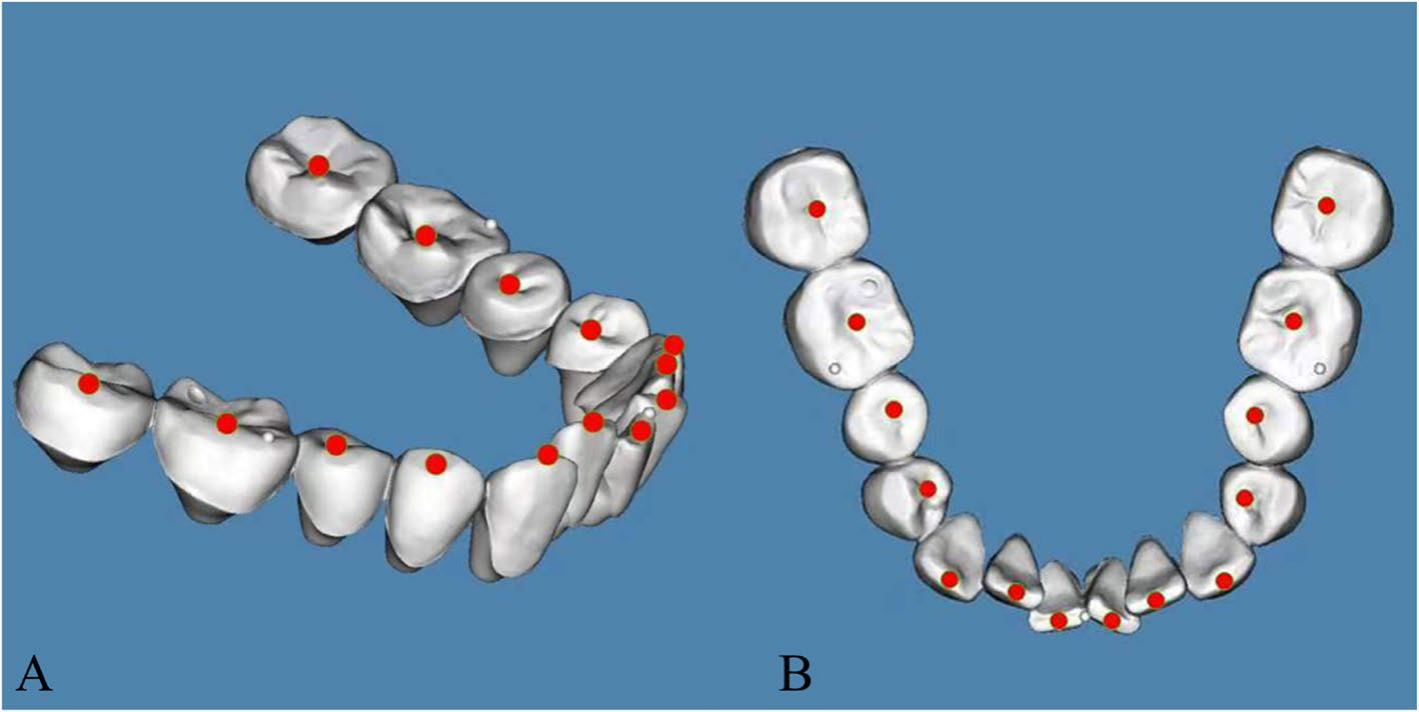
Measurement. The three-dimensional position of each crown was measured. Crown points were set at the middle fossa of molars or premolars, the cusp of canines, and the midpoint of the incisal edge. **A** Isometric view. **B** Top view

**Fig. 8:**
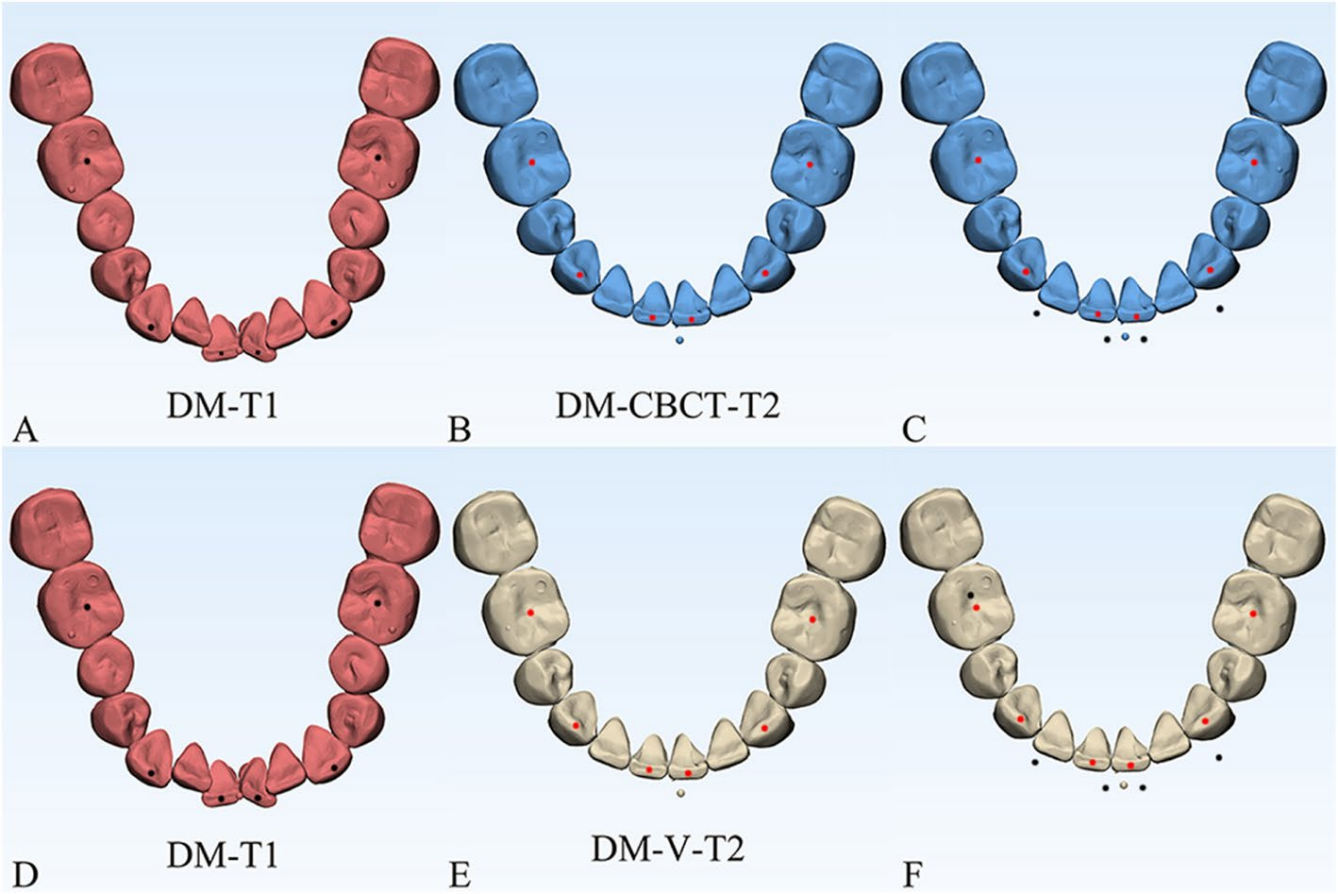
3-D measurement. Six landmarks were placed on the middle of incisal edges of the lower middle incisors, canine cusp tips and the central fossa of first molars as black landmarks in DM-T1 models (**A**, **D**) and as red landmarks in DM-CBCT-T2 and DM-V-T2 models (**B**, **E**). Measurement of the 3-D distance between pretreatment (DM-T1) and posttreatment (DM-CBCT-T2 and DM-V-T2) were both calculated. Landmarks of DM-T1 might be covered by DM-CBCT-T2 or DM-V-T2 (**C**,** F**)

1. Marker placement: In 3-Shape Appliance Analyze (v1.9.2.3, 3-Shape Inc., Copenhagen, DK), the branches of vessels in the bilateral lingual side of T1 mandibular DMs could be easily recognized. The markers (sphere in 1 mm diameter) were placed manually along the distribution of vessels (Fig. 1A). The same procedures were repeated in T2 DMs (Fig. 1B). All the markers were merged with DMs (DM-T1 and DM-T2) and exported as standard triangulated language (STL) format (Fig. 1C, D).
2. CBCT registration: T1 and T2 CBCT images were imported into Dolphin Imaging (v11.95, Dolphin Imaging and Management Solutions Inc., Chatsworth, CA, USA) and registered together with the mandibular body and part of the ramus as the reference region (Fig. 2), which was considered as voxel-based registration. The registered CBCT-T1 and CBCT-T2 were then exported in DICOM format and converted to STL format (Fig. 2C) by Mimics Research (version 21.0, Materialise N.V., Technologielann, Leuven, BE)
3. Settle the position of DM-T1: DM-T1 acquired from STEP 1 was registered to CBCT-T1 acquired from STEP 2 by the morphology of dentition as a reference area in 3-matic Research (v13.0, Materialise N.V., Technologielann, Leuven, BE). Then, the position of DM-T1 was fixed (Fig. 3A).
4. CBCT-based DMs superimposition (CBCT group): Because the position of DM-T1 was fixed in CBCT-T1, and the CBCT-T2 had already been registered over CBCT-T1, we only needed to register the DM-T2 over CBCT-T2 as STEP 3 did (Fig. 3B), and then we could obtain the DMs superimposed by CBCT voxel-based registration: DM-CBCT-T2 (Fig. 3C).
5. Vessel-based DMs superimposition (Vessel group): DM-T2 was superimposed on DM-T1 acquired from STEP 3 with the vessel markers as the reference area (Fig. 4A). Finally, we acquired the DMs superimposed by the characteristics of vessels: DM-V-T2. Now, we can visually recognize the differences between the 2 superimposition methods (Fig. 4B, C).
6. Segmentation: For further study of tooth movement during the treatment, data acquired from the above needed to be further processed. In the 3-Shape Appliance Analyze, pre- and posttreatment mandibular DMs were segmented to the tooth models (Fig. 5A).
7. Establish the coordinate system: The tooth models obtained in STEP 6 were aligned with DM-T1, DM-CBCT-T2, and DM-V-T2. These scanning models were then transformed into dentition models (Fig. 5B). Three spheres were positioned at the mesial-buccal cusp of the bilateral mandibular first molars and the midpoint of the bilateral central incisors on DM-T1. These spheres were duplicated on DM-CBCT-T2 and DM-V-T2 to ensure the consistency of the coordinate system (Fig. 6).
8. Measurement: The crown points were set at the midpoint of the incisal ridge, cups of canines and the central fossa of molars and premolars (Fig. 7). The 3-D coordinate values of each tooth crown could be determined as (X, Y, Z) in the system (X represented the horizontal tooth movement, Y represented the vertical tooth movement, Z represented the sagittal tooth movement). In addition, 3-D measurements of bilateral lower first molars, canines, incisors movement between T1 and T2 in 2 groups were also calculated. Six landmarks were placed on the middle of incisal edges of the lower middle incisors, canine cusp tips and the central fossa of first molars as black landmarks in DM-T1 models (Fig. 8A, D) and as red landmarks in DM-CBCT-T2 and DM-V-T2 models (Fig. 8B, C, E and F).

The sample size of this study was calculated by PASS (v15.0, NCSS, LLC. Kaysville, Utah, USA). Wilcoxon’s Sign Rank Test was performed to compare the measurement results obtained from two distinct superimposition methods. Statistical significance was established at a *P* value less than 0.05, provided the data met the standard distribution criteria and homogeneity of variance. The standard deviation of paired differences was set as 0.45 based on the pre-experiment results, the mean of paired differences was set as 0.3 based on the level of clinical acceptability, the testing power was set as 0.9, α = 0.05, and the nominal size was 26.

SPSS Statistics (v25.0, SPSS Inc., Chicago, IL, USA), was used to process all the statistical analyses. Rater 1 determined the intrarater reliability, repeated all the procedures of superimposition and measurement after 1 week and then tested it using the intraclass correlation coefficient (*ICC*). This study was based on a single rater (*k* = 1) using absolute agreement and the 2-way mixed-effects model at a 95% confidence interval. As for the interrater reliability, rater 2 repeated the above steps to register the CBCT and DMs; the measurement results were compared by interclass correlation coefficient, based on a mean rating (*k* = 2), using absolute agreement and the 2-way random-effects model at a 95% confidence interval.

In addition, two independent T test was used to compare the 3-D measurement of 2 groups (significance established at *P* = 0.05). The values of root mean square (*RMS*) between DM-CBCT-T2 and DM-V-T2 were also calculated by part comparison analysis in 3-Matic Research.

## Results

**Fig. 9:**
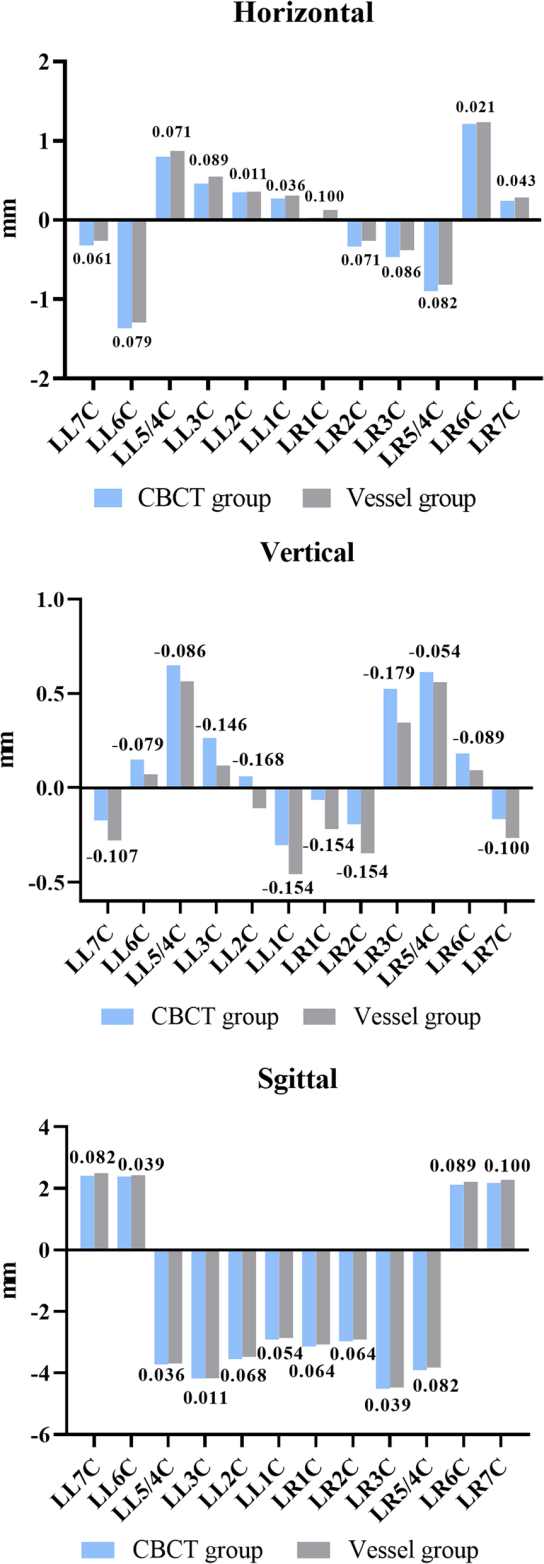
The three-dimensional tooth movement of crown in two superimposition methods was demonstrated, as well as the differences between ‘DM-CBCT-T2’ and ‘DM-V-T2’ (calculated by subtracting ‘DM-CBCT-T2’ from ‘CBCT-V-T2’)

No significant differences were found between the measurements of the 2 superimposition methods in the horizontal, vertical, and sagittal directions in the crown (*P* > 0.05) (Table 1). The median, 25th percentile and 75th percentile of tooth movement in each direction are shown in Table 1. The mean differences between the 2 methods were also calculated (DM-CBCT-T2 subtracted from CBCT-V-T2) and shown in Fig. 9. In the crown, the maximum differences between the 2 methods were + 0.100 mm in the horizontal direction, − 0.179 mm in the vertical direction and + 0.100 mm in the sagittal direction.

**Table 1:** Wilcoxon’s Sign Rank Test for comparing the tooth movement in three-dimensional direction of each crown point in two superimposition methods (*P* > 0.05)

Samples (*n* = 28)	Horizontal				Vertical					Sagittal		
	Median (P_25_, P_75_)		*Z*	*P*	Median (P_25_, P_75_)		*Z*	*P*		Median (P_25_, P_75_)	*Z*	*P*
	CBCT Group	Vessel Group			CBCT Group	Vessel Group			CBCT Group	Vessel Group		
LL7R	−0.60 (−1.20, − 0.13)	− 0.50 (− 1.08, − 0.10)	−1.433	0.152	− 0.25 (− 0.78, 0.00)	−0.30 (− 1.08, − 0.03)	−1.434	0.152	3.05 (1.93, 3.80)	3.30 (1.88, 3.98)	−1.884	0.060
LL6R	−1.30 (− 1.78, − 0.83)	−1.35 (− 1.80, − 0.73)	−1.808	0.071	0.20 (− 0.30, 0.70)	0.10 (− 0.38, 0.58)	− 1.886	0.059	3.20 (1.80, 3.88)	3.30 (1.93, 3.80)	−1.466	0.143
LL5/4R	1.15 (0.65, 1.48)	1.20 (0.70, 1.58)	−1.759	0.079	0.90 (0.00, 1.30)	0.80 (−0.10, 1.18)	−1.913	0.056	−3.40 (−4.63, − 2.50)	− 3.20 (− 4.50, − 2.23)	− 1.674	0.094
LL3R	1.40 (0.83, 2.20)	1.35 (0.90, 2.45)	− 1.410	0.159	0.20 (− 0.78, 0.70)	− 0.05 (− 0.78, 0.58)	− 1.860	0.063	−3.20 (− 4.35, − 2.63)	−3.50 (− 4.20, − 2.53)	− 0.937	0.349
LL2R	0.90 (0.43, 1.93)	0.80 (0.33, 1.98)	−0.363	0.717	−0.30 (− 1.18, 0.43)	− 0.60 (− 1.38, 0.50)	−1.878	0.060	− 2.70 (− 3.08, − 2.05)	− 2.65 (− 2.90, − 1.90)	− 1.947	0.052
LL1R	0.20 (− 0.28, 0.80)	0.20 (− 0.25, 0.83)	− 0.738	0.046	− 0.55 (− 1.28, − 0.03)	−0.80 (− 1.38, − 0.10)	−1.686	0.092	− 2.10 (− 2.50, − 1.15)	−1.95 (− 2.50, − 1.13)	−1.456	0.145
LR1R	− 0.30 (− 0.88, 0.20)	− 0.15 (− 0.60, 0.30)	−1.788	0.074	− 0.20 (− 1.35, 0.08)	−0.40 (− 1.48, 0.10)	−1.630	0.103	− 2.30 (− 2.80, − 1.35)	−2.30 (− 2.78, − 1.25)	−0.902	0.367
LR2R	−1.00 (− 1.70, − 0.05)	−0.90 (− 1.83, − 0.10)	−0.769	0.442	−0.50 (− 1.18, 0.10)	−0.75 (− 1.10, 0.05)	−1.843	0.065	−2.50 (− 3.08, − 1.80)	−2.50 (− 2.98, − 1.65)	− 1.489	0.136
LR3R	−1.30 (− 1.78, − 0.63)	−1.15 (− 1.50, − 0.70)	−1.585	0.113	0.85 (0.23, 1.10)	0.70 (0.35, 1.10)	−1.860	0.063	− 3.45 (− 3.95, − 2.63)	−3.50 (− 3.88, − 2.55)	− 1.825	0.068
LR5/4R	−0.85 (− 1.48, − 0.08)	−0.90 (− 1.48, − 0.08)	−0.513	0.608	0.90 (0.23, 1.20)	0.70 (0.35, 1.10)	−1.801	0.072	−3.25 (−4.05, − 2.60)	−3.30 (− 4.00, − 2.30)	−1.744	0.081
LR6R	1.30 (0.83, 1.90)	1.40 (0.90, 1.88)	−1.216	0.224	0.10 (−0.10, 0.40)	0.05 (−0.30, 0.38)	−1.891	0.059	2.95 (2.08, 3.58)	3.05 (2.10, 3.68)	−0.692	0.489
LR7R	0.70 (0.53, 1.18)	0.75 (0.40, 1.20)	−0.053	0.958	−0.50 (− 0.80, − 0.03)	−0.55 (− 0.98, − 0.03)	−1.833	0.067	2.90 (1.33, 3.73)	3.10 (1.50, 3.75)	−1.024	0.306

Tooth movement in horizontal (right-left), vertical (superior-inferior) and sagittal (anterior-posterior) of each crown are presented as median (*P*_25_, *P*_75_)

*LL7C* lower left second molar crown, *LL6C* lower left first molar crown, *LL5/4C* lower left premolar crown, *LL3C* lower left canine crown, *LL2C* lower left lateral incisor crown, *LL1C* lower left incisor crown, *LR1C* lower right incisor crown, *LR2C* lower right lateral incisor crown, *LR3C* lower right canine crown, *LR5/4C* lower right premolar crown, *LR6C* lower right first molar crown, *LR7C* lower right second molar crown

The intraclass and interclass correlation coefficient results showed excellent intrarater reliability (e.g., above 0.891 in the crown, Table 2) and interrater agreement (e.g., above 0.888 in the crown, Table 3).

**Table 2:** Intrarater agreement of rater 1 for the three-dimensional coordinate values of the crown

Intrarater agreement (crown)									
Samples (*n* = 28)	Horizontal			Vertical			Sagittal		
	*ICC*	95% Confidence interval		*ICC*	95% Confidence interval		*ICC*	95% Confidence interval	
		Lower bound	Upper bound		Lower bound	Upper bound		Lower bound	Upper bound
LL7C	0.990	0.979	0.995	0.944	0.864	0.975	0.963	0.923	0.983
LL6C	0.975	0.948	0.988	0.950	0.874	0.978	0.932	0.860	0.968
LL5/4C	0.975	0.946	0.988	0.965	0.926	0.984	0.949	0.892	0.976
LL3C	0.968	0.932	0.985	0.976	0.946	0.989	0.965	0.914	0.985
LL2C	0.977	0.950	0.989	0.985	0.964	0.994	0.977	0.926	0.991
LL1C	0.955	0.903	0.979	0.983	0.964	0.992	0.970	0.879	0.989
LR1C	0.923	0.843	0.964	0.986	0.969	0.994	0.968	0.831	0.989
LR2C	0.907	0.811	0.956	0.984	0.966	0.993	0.965	0.856	0.987
LR3C	0.966	0.928	0.984	0.974	0.939	0.989	0.966	0.886	0.987
LR5/4C	0.969	0.934	0.985	0.891	0.780	0.948	0.969	0.934	0.986
LR6C	0.980	0.955	0.991	0.891	0.765	0.949	0.952	0.898	0.977
LR7C	0.957	0.907	0.980	0.933	0.836	0.971	0.952	0.898	0.977

*ICC* interclass correlation coefficient. Tooth movement in horizontal (right-left), vertical (superior-inferior) and sagittal (anterior-posterior) directions

**Table 3:** Interrater agreement of rater 1 and rater 2 for the three-dimensional coordinate values of the crown

Interrater agreement (crown)									
Samples (*n* = 28)	Horizontal			Vertical			Sagittal		
	*ICC*	95% Confidence interval		*ICC*	95% Confidence interval		*ICC*	95% Confidence interval	
		Lower bound	Upper bound		Lower bound	Upper bound		Lower bound	Upper bound
LL7C	0.988	0.975	0.995	0.954	0.903	0.978	0.965	0.926	0.984
LL6C	0.978	0.954	0.990	0.945	0.886	0.974	0.934	0.863	0.969
LL5/4C	0.975	0.947	0.988	0.964	0.925	0.983	0.957	0.902	0.980
LL3C	0.964	0.924	0.983	0.973	0.924	0.989	0.968	0.921	0.986
LL2C	0.978	0.953	0.990	0.984	0.955	0.994	0.980	0.935	0.992
LL1C	0.962	0.921	0.982	0.983	0.962	0.992	0.975	0.879	0.992
LR1C	0.945	0.885	0.974	0.987	0.970	0.994	0.969	0.873	0.989
LR2C	0.918	0.832	0.961	0.983	0.962	0.992	0.966	0.864	0.988
LR3C	0.960	0.916	0.981	0.973	0.936	0.988	0.955	0.882	0.981
LR5/4C	0.964	0.923	0.983	0.898	0.794	0.951	0.965	0.926	0.984
LR6C	0.988	0.973	0.995	0.888	0.768	0.947	0.955	0.905	0.979
LR7C	0.959	0.908	0.981	0.917	0.798	0.964	0.962	0.921	0.982

*ICC* interclass correlation coefficient. Tooth movement in horizontal (right-left), vertical (superior-inferior) and sagittal (anterior-posterior) directions

**Fig. 10:**
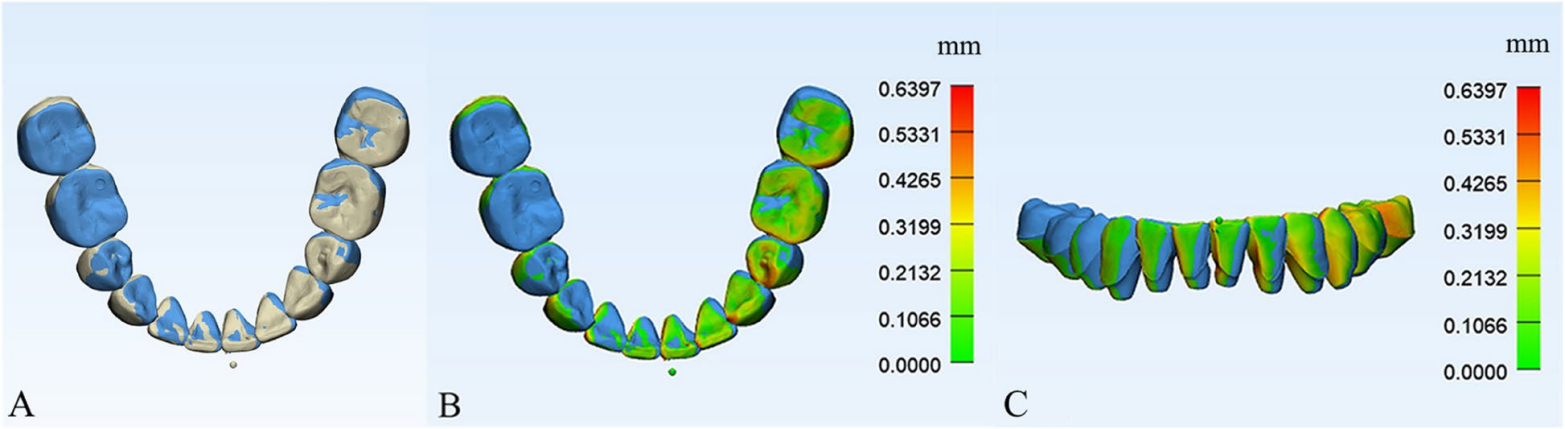
Part comparison analysis. **A** Top view of DM-CBCT-T2 (blue) and DM-V-T2 (yellow). **B, C** Top view and frontal view of part comparison analysis of two parts, the color bar (range from green to red) shows the differences

No statistical significance was found in the 3-D measurement results between the 2 groups (Table 4, *P* > 0.05). The *RMS* values between DM-CBCT-T2 and DM-V-T2 are shown in Table 5**.**All the *RMS* values were lower than 0.5 mm. The results of part comparison analysis showed in Fig. 10, the color of DM-V-T2 surface, which ranged from green to red, presented for the differences of two parts.

**Table 4:** Two independent samples T test showed that there was no statistical significance of 3-D measurements between CBCT group and Vessel group (*P* > 0.05)

	CBCT Group	Vessel Group	*P* value	95% Confidence Interval
	Mean ± SD	Mean ± SD		
LL6	3.10 ± 1.20	3.13 ± 1.22	0.802	(−0.675, 0.619)
LL3	4.65 ± 1.36	4.52 ± 1.26	0.754	(− 0.568, 0.839)
LL1	3.46 ± 1.17	3.33 ± 1.14	0.921	(− 0.486, 0.751)
LR1	3.71 ± 1.24	3.58 ± 1.20	0.946	(− 0.525, 0.784)
LR3	5.01 ± 1.71	4.87 ± 1.66	0.905	(− 0.763, 1.038)
LR6	2.87 ± 1.08	2.98 ± 1.06	0.858	(− 0.684, 0.465)

LL6, 3-D measurement of lower left first molars between T1 and T2 models

LL3, 3-D measurement of lower left canines between T1 and T2 models

LL1, 3-D measurement of lower left middle incisors between T1 and T2 models

LR1, 3-D measurement of lower right middle incisors between T1 and T2 models

LR3, 3-D measurement of lower right canines between T1 and T2 models

LR6, 3-D measurement of lower right first molars between T1 and T2 models

**Table 5:** The mean, standard deviation and root mean square values between DM-CBCT-T2 and DM-V-T2

Samples (*n* = 28)	Part comparison analysis		
	Mean	SD.	*RMS*
Patient 1	0.27	0.17	0.32
Patient 2	0.35	0.23	0.42
Patient 3	0.24	0.16	0.29
Patient 4	0.31	0.22	0.38
Patient 5	0.26	0.17	0.31
Patient 6	0.25	0.15	0.29
Patient 7	0.35	0.23	0.42
Patient 8	0.32	0.26	0.41
Patient 9	0.15	0.10	0.18
Patient 10	0.08	0.05	0.09
Patient 11	0.27	0.22	0.35
Patient 12	0.13	0.11	0.18
Patient 13	0.24	0.16	0.29
Patient 14	0.23	0.17	0.28
Patient 15	0.30	0.20	0.36
Patient 16	0.15	0.14	0.20
Patient 17	0.29	0.19	0.35
Patient 18	0.26	0.19	0.32
Patient 19	0.27	0.20	0.34
Patient 20	0.26	0.16	0.31
Patient 21	0.19	0.13	0.23
Patient 22	0.19	0.13	0.23
Patient 23	0.13	0.09	0.16
Patient 24	0.19	0.13	0.23
Patient 25	0.19	0.12	0.23
Patient 26	0.22	0.14	0.26
Patient 27	0.09	0.06	0.11
Patient 28	0.24	0.16	0.29

SD standard deviation, *RMS* root mean square

**Fig. 11:**
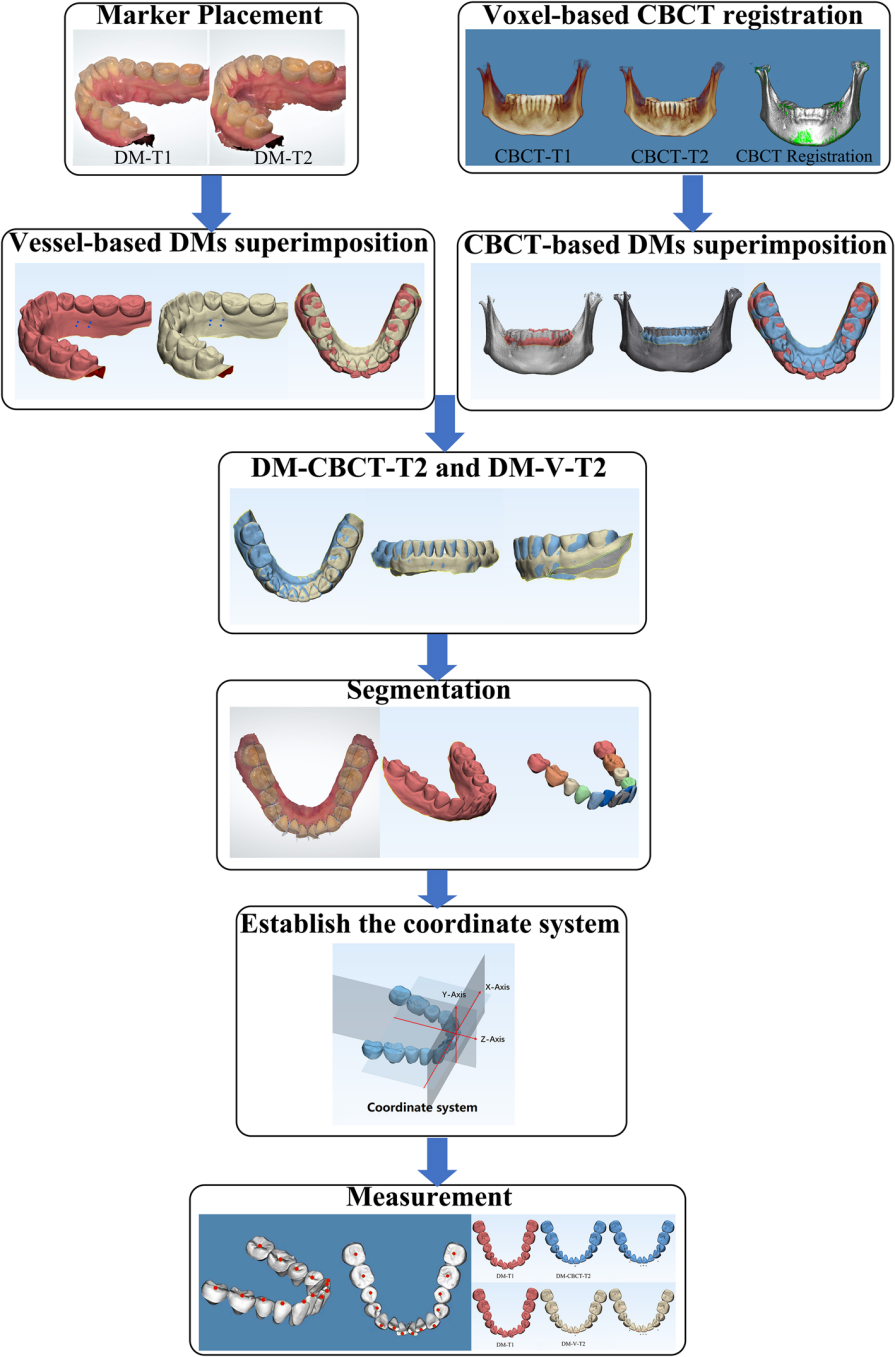
Flowchart of the steps for the mandibular digital model (DM) registration method

A flow chart depicting the steps for the method of mandibular DM superimposition is provided in Fig. 11.

## Discussion

Three-dimensional digital models are widely used for clinical practice because it can record intraoral tissue without radiation efficiently [25]. Meanwhile, DMs are potential tools for sequential orthodontic treatment assessment. However, researchers have not reached a definite conclusion about the evident and stable anatomical structure in mandibular DMs thus far. Thus, it is urgent to find a method to superimpose mandibular DMs efficiently and accurately, especially in extraction cases.

The mucogingival line was considered as a stable region, and its morphological features were used as a reference for mandibular DMs in non-extraction cases [26–29]. Some researchers have reported that the vessels of the fundus in the different periods were available for superimposing the images [23, 24]. Our study also uncovered an extensive network of vessels on both bilateral lingual sides of the mandibular DMs. Therefore, we proposed the hypothesis of superimposing mandibular DMs by the characteristics of vessels. This study aimed to validate the availability and reliability of superimposing full-color mandibular digital models through the morphological characteristics of vessels.

Presently, few studies have reported methods of superimposing mandibular DMs. A previous study proved that the mandibular torus was a potential reference area of superimposition in the extraction case, and the mandibular alveolar surfaces seem unstable because of active remodeling [22]. Dai attempted to preliminarily superimpose the mandibular DMs through the palatal rugae of the maxillary and the occlusion relationship, then adjust the mandibular rotation and translation by cephalometric film created from CBCT images, and compared the results with the CBCT surface-based mandibular DMs superimposition [19]. No significant differences were found except for the horizontal movement of incisors and canines. However, these two methods could not adjust the rotation and translation in the horizontal direction in the light of cephalometric films. Besides cephalometric radiographs have several disadvantages, such as overlapping structures, magnification and distortion of the image. In our study, the outcome of tooth 3-D movement was compared to that of CBCT voxel-based DM superimposition, which was regarded as the benchmark standard.

We proposed that the method can superimpose mandibular models directly with the lingual vessels as anatomical reference areas, which is simpler and more effective than the other methods. This method not only reduces radioactive damage to patients but also accurately measures the three-dimensional movement of each tooth in different time periods. The past researches have confirmed that the mucogingival junction could stay relatively stable during orthodontic treatment [26, 30, 31]. In some cases, the high frenal attachment of buccal frenum might tract the periodontal tissue and lower the stability of this area [32, 33], which were not suitable for digital model superimposition. Thus, we chose the vessels in the lingual sides of mandible, near the level of mucogingival junction as the potential reference region for superimposition. To further illustrate the accuracy of the method, we compared each pair of DMs from the CBCT group and Vessel group by the function of part comparison analysis in 3-Matic Research, which visually displayed the differences (Fig. 10). The *RMS* values were all lower than 0.5 mm (Table 5). The minor differences between the Vessel group and the CBCT group were clinically acceptable and showed no statistical significance. However, the active reconstruction of alveolar bone and significant movement of teeth in extraction cases posed challenges for superimposition. To reduce the errors, all reference points avoided settling at the anterior or posterior active remodeling areas. All markers were positioned in the lingual sides, between the premolars and first molar, near the level of mucogingival junction, and dispersed to the greatest extent possible.

There were some inadequacies for improvement of this method. First, scanning the lingual tissue in the mandible may prove difficult due to the unique shape of the tongue and the depth of the oral cavity in individual patients. Second, the vessel markers were placed manually and limited in the region of the premolars to the first molar, so the markers should be dispersed as widely as possible.

## Conclusions

The courses of vessels in the bilateral lingual sides of full-color mandibular DMs can remain stable in adult cases during orthodontic extraction treatment and be a potential reference region for mandibular digital model superimposition. This method provides an efficient way to evaluate tooth movement and decreases the risk of radiation exposure to patients.

## Data Availability

The data that support the findings of this study are available from the corresponding author upon reasonable request.

## Abbreviations

CBCT: Cone-beam computed tomography
ALARA: As low as reasonably achievable
DMs: Digital models
FOV: Field of view
LL7C: Lower left second molar crown
LL6C: Lower left first molar crown
LL5/4C: Lower left premolar crown
LL3C: Lower left canine crown
LL2C: Lower left lateral incisor crown
LL1C: Lower left incisor crown
LR1C: Lower right incisor crown
LR2C: Lower right lateral incisor crown
LR3C: Lower right canine crown
LR5/4C: Lower right premolar crown
LR6C: Lower right first molar crown
LR7C: Lower right second molar crown
*ICC*: Intraclass or interclass correlation coefficient
SD: Standard deviation
*RMS*: Root mean square
